# Parthenolide attenuated the endometriosis-like lesions by activating autophagy and suppressing NLRP3 inflammasome activity

**DOI:** 10.22038/ijbms.2025.90575.19521

**Published:** 2026

**Authors:** Luhongyuan Jin, Tingting Fu, Chi Chi, Jiayi Zhou, Jun Lin, Wenjie Hou

**Affiliations:** 1 Department of Gynecology and Obstetrics, The Fourth Affiliated Hospital of Soochow University, Suzhou 215000, China; 2 Department of Orthopedics, The Fourth Affiliated Hospital of Soochow University, Suzhou 215000, China; 3 Nanjing University of Chinese Medicine, Nanjing 210003, China

**Keywords:** Autophagic, Endometriosis, Inflammasomes, Inflammatory response, NLRP3 protein, Parthenolide

## Abstract

**Objective(s):**

Parthenolide (PTL) has significant anti-inflammatory and immunomodulatory effects, but its regulatory mechanisms in endometriosis (EMs) remain unclear. This study aimed to systematically evaluate the effects of PTL on cellular models and a murine EMs model, with a focus on its regulatory roles in autophagy and the NLRP3 inflammasome pathway.

**Materials and Methods:**

Human monocytic leukemia THP-1 cells, murine immortalized bone marrow-derived macrophages, and 30 female C57BL/6 mice were used. Autophagy-related proteins (Beclin1, LC3, p62) and inflammasome components (NLRP3, ASC, caspase-1) were detected by Western blotting, and the activation of the AMPK/ULK1 signaling pathway was assessed after treating with PTL at a concentration of 10 mg/ml for 1 hr. A murine EMs model was established by peritoneal implantation, followed by intraperitoneal injections of PTL (10 mg/ml). Immunohistochemical staining was performed to detect the expression of NLRP3, caspase-1, IL-1β, and GSDMD in ectopic lesions.

**Results:**

*In vitro*, PTL (10 mg/ml) significantly inhibited the activation of NLRP3, caspase-1-p20, IL-1β, and GSDMD, while increasing the phosphorylation levels of Beclin1 and AMPK/ULK1, and decreasing the expression of p62 and LC3, indicating enhanced autophagic flux. *In vivo*, PTL treatment markedly reduced the number, surface area, and weight of ectopic lesions in mice, and significantly suppressed the expression of inflammatory proteins in the lesions.

**Conclusion:**

PTL exerts its therapeutic effect on EMs by simultaneously activating autophagy through the AMPK/ULK1 signaling pathway and inhibiting the NLRP3 inflammasome and its downstream effectors.

## Introduction

Endometriosis (EMs) is a chronic gynecological condition defined by the ectopic presence of endometrial tissue outside the uterine cavity. It is primarily manifested by chronic pelvic pain and infertility (1, 2). The main therapeutic approaches for EMs are hormone-based treatments and surgery. Gonadotropin-releasing hormone (GnRH) agonists and progestins are frequently used to manage lesion growth and alleviate pain (3). These treatments often provide short-term relief, but long-term use may lead to adverse effects, such as reduced bone density. 

Recently, research has increasingly focused on non-hormonal treatments, particularly those targeting specific molecular pathways (4). Elucidating these molecular mechanisms, especially the interactions among inflammation, immune regulation, and autophagy, provides a promising basis for the development of novel therapies (5, 6).

Abnormal activation of inflammasomes, particularly NLRP3, has been implicated in the pathogenesis of EMs (7). Concomitantly, several autophagy-related genes, including *Beclin-1* and *LC3*, as well as key regulatory pathways such as PI3K/AKT/mTOR, HIF-1α, NF-κB, and Rho/ROCK are known to be involved in disease progression (8, 9). Increasing evidence suggests that enhancing autophagic activity can attenuate NLRP3-driven inflammatory responses, thereby potentially delaying EM progression (10). 

Autophagy negatively regulates NLRP3 inflammasome activation by promoting the degradation of its components. This process limits the downstream production of pro-inflammatory cytokines. In patients with EMs, impaired autophagy contributes to sustained inflammasome activation. This leads to exacerbated inflammation, increased tissue damage, and enhanced survival and proliferation of ectopic endometrial cells (11). Notably, autophagic flux is markedly reduced in EM tissues, while NLRP3 activity is aberrantly elevated (12). These findings highlight the therapeutic potential of targeting NLRP3 inflammasome activity, either directly or via modulation of autophagy, as a novel strategy for EMs management.

New evidence shows that parthenolide (PTL) can reduce inflammation by targeting the NLRP3 inflammasome and dampening proinflammatory signals (13). It is unknown, however, if PTL has these effects in EMs. The role of autophagy in PTL’s action on NLRP3 activity has also not been fully explored. Without this knowledge, our understanding of PTL’s therapeutic relevance for EM is incomplete. Both disrupted autophagy and abnormal NLRP3 activation are important in EMs development. Therefore, this study explores PTL’s dual effects on autophagy and NLRP3 inflammasome suppression. Clarifying the interplay between autophagy and NLRP3 may reveal new intervention targets. This could support PTL as a non-hormonal treatment candidate for EMs. Targeting both pathways might help develop therapies tailored to immune and autophagy issues in EMs. In humans, ethical concerns limit studies to comparing drug effects from samples obtained in repeated surgeries. Because of this, we created a mouse model to evaluate PTL’s therapeutic effects in EMs. 

## Materials and Methods

### Cell culture

The human monocytic cell line THP-1 was obtained from the Cell Bank of the Chinese Academy of Sciences. Cells were cultured in RPMI 1640 medium (Cat No.:11875093, Gibco) with 10% fetal bovine serum (FBS, Cat No.:10099141, Gibco) and 1% penicillin-streptomycin (Cat No.:15140122, Gibco) at 37 °C in a humidified 5% CO₂ atmosphere. To induce macrophage differentiation, THP-1 cells were treated with 100 nM PMA (Sigma-Aldrich, Cat No.: P8139) for 24 hours. After washing with PBS, they were incubated in fresh medium without PMA for another 24 hr.

Mouse bone marrow-derived macrophages (BMDMs) were isolated from femurs and tibias of 8- to 10-week-old C57BL/6J mice using established protocols (14). Cells were cultured in RPMI 1640 with 20% FBS and 20 ng/ml recombinant mouse M-CSF (Peprotech, Cat No.: 315-02) at 37 °C in 5% CO₂ for 7 days. The medium was changed every two days. Ethics approval was obtained from the Animal Ethics Committee of Soochow University (Approval No: SUDA20250720A05).

### Grouping

Differentiated THP-1 macrophages or BMDMs (1×10⁵) were first primed with 1 μg/ml lipopolysaccharide (LPS, Sigma-Aldrich, Cat. No.: L4391) for four hours to induce NLRP3 and pro-IL-1β expression, to activate the NLRP3 inflammasome. Cells were then stimulated with 5 mM ATP (Sigma-Aldrich, Cat. No.: A2383) for 30 min, followed by one hour to promote NLRP3 inflammasome assembly and activation. To investigate the regulatory effects of PTL (MedChemExpress, Cat. No.: HY-N0141, Purity 99.91%) on NLRP3 inflammasome activation and autophagy, cells primed with LPS (1 μg/ml) and subsequently stimulated with ATP (5 mM) were treated with PTL at concentrations of 5, 10, and 15 mg/ml, respectively. 

Cells that received no treatment served as the control group, while those subjected to LPS and ATP were designated as the LPS+ATP group.

After treatment, the cell lysates and supernatant were collected for further analysis. The cell lysates were utilized to assess the expression of NLRP3, ASC, pro-caspsease-1/caspase-1-p20, pro-IL-1β, LC3, p62, beclin1, and components of the AMPK/ULK1 pathway, including the phosphorylation. The supernatant was utilized to determine the release of extracellular inflammatory factors such as mature IL-1β and caspase-1-p20. All these tests were performed at least in triplicate. 

### Immunofluorescence staining

Cells were seeded in 24-well plates and cultured overnight, and divided into the control group, the LPS+ATP group, and the LPS+ATP+PTL (10 mg/ml) group. For the inflammasome activation, cells were pretreated with LPS (1 μg/ml) for four hours, then stimulated with ATP (5 mM) for 45 min and incubated at 37 °C. In the PTL group, PTL (10 mg/ml) was added one hour before ATP stimulation. After treatment, cells were washed twice with pre-cooled PBS and then fixed with 4% paraformaldehyde for 30 min. After fixation, cells were permeabilized with 0.3% Triton X-100 for 15 minutes and blocked with 5% bovine serum albumin (BSA) for one hour. ASC primary antibody (Cat. No.: ab155970; 1 200, Abcam) was then added and incubated overnight at 4 °C. After approximately 24 hr, the cells were washed with PBS three times, and the secondary antibody labeled with Alexa Fluor 594 (Cat. No.: ab150080; 1 500, Abcam) was added and incubated at room temperature in the dark for one hour. Nuclear staining was performed with DAPI staining solution for 5 min. Subsequently, the cells were sealed with an anti-fluorescence quenching sealing agent. All immunofluorescence experiments were performed with three independent biological replicates. For each sample, five non-overlapping fields of view were randomly selected for observation and image acquisition to ensure the representativeness of the results. Inflammasome assembly was assessed by observing the transition of ASC protein from a diffuse distribution to punctate aggregates. Images were acquired using a Leica DMi8 fluorescence microscope. Quantitative analysis was performed using ImageJ software. Additionally, the percentage of ASC-positive cells (ASC-positive cells/total DAPI-positive cells) was calculated.

### Assessment of cell viability by Calcein-AM/PI staining

Cells (1×10⁵) were seeded into 12-well plates and cultured for 12 hr. After incubation, the culture medium was carefully removed, and the cells were gently washed with pre-warmed PBS to eliminate the residual media. Cell viability staining was then performed using the Calcein/PI Cell Viability/Cytotoxicity Assay Kit (Beyotime Biotechnology, Cat. No.: C2015), following the manufacturer’s instructions. Calcein-AM (2 μM) was used to stain live cells, while propidium iodide (PI, 5 μM) was used to stain the dead cells. A total of 200 μl of working solution was added to each well, followed by incubation at 37 °C in the dark for 1530 min. After staining, excess dye was removed by washing with PBS. Fluorescent images were captured using a fluorescence microscope (Leica DMi8). Green fluorescence from Calcein-AM-labeled live cells was detected using the CH2 channel with an excitation wavelength at 495 nm and an emission wavelength at 515 nm. The red fluorescence from PI-labeled dead cells was detected using the CH3 channel with an excitation wavelength at 535 nm and an emission wavelength at 617 nm. The quantitative analysis was performed using the ImageJ software.

### Preparation and stimulation of BMDMs from Atg5fl/fl and LysM-Cre conditional knockout mice

Femurs and tibiae were obtained from Atg5fl/flLysM-Cre− and Atg5fl/flLysM-Cre+ mice (8 weeks, female) that were kindly bequeathed by Professor Jun Lin at Soochow University. Using the LysM-Cre system, Atg5 is specifically deleted in myeloid cells (mainly macrophages and neutrophils) in Atg5fl/fl LysM-Cre+ mice, resulting in autophagy deficiency. Atg5fl/fl LysM-Cre− mice serve as controls with normal autophagy. The modeling process was established based on the previous protocols(15). Bone marrow cells were collected by flushing femurs and tibias, followed by filtration through a 70 μm filter. The cells were cultured in DMEM supplemented with 10% FBS and 1% penicillin-streptomycin (Gibco, Cat No.: 11995), along with 20 ng/ml MCSF (PeproTech, Cat No.: 315-02). Cells were plated at 1×10⁶ per well in 6-well plates and maintained for 7 days. Afterwards, the cells were treated with LPS (1µg/ml, for 4 hr), LPS+ATP (5 mM, for 45 min), and LPS+ATP+PTL (10 mg/ml for 1 hr), followed by determination of the expression of IL-1β pro, IL-1β, caspase pro, and caspase-1-p20 using Western blot analysis. 

### Pharmacological inhibition of the autophagy and secretory pathway

To investigate the role of autophagy and the classical secretory pathway in PTL-mediated regulation of inflammation, pharmacological inhibitors 3-Methyladenine (3-MA) and Brefeldin A (BFA) were employed. The following groups were set, including the control group, the LPS group, the LPS+ATP group, the LPS+ATP+PTL group, the LPS+ATP+PTL+3-MA group, and the LPS+ATP+PTL+BFA group, respectively. Cell lysates were prepared using RIPA buffer supplemented with protease and phosphatase inhibitors, and centrifuged at 12,000 rpm for 15 minutes at 4  °C. The resulting lysates and supernatants were collected for subsequent Western blot and cytokine analysis. Western blot analysis was performed using ImageJ. 

### Western blot analysis

Total cellular proteins were extracted using RIPA buffer, followed by protein concentration determination using the BCA method. Then the protein samples collected from the supernatant were separated by SDS-PAGE gel and transferred to PVDF membranes. Membranes were probed with the primary antibodies against full-length GSDMD (GSDMD-FL) (Cat. No.: ab209845; 1: 1000, Abcam) and its N-terminal cleaved fragment (GSDMD-NT) (Cat. No.: ab215191; 1: 1000, Abcam), NLRP3 (Cat. No.: ab263899; 1: 1000, Abcam), caspase-1 (Cat. No.: ab207802; 1: 1000, Abcam), P20 (Cat. No.: ab179515; 1: 1000, Abcam), IL-1β pro (Cat. No.: ab2105; 1: 1000, Abcam), IL-1β (Cat. No.: ab9722; 1: 1000, Abcam), ASC (Cat. No.: ab155970; 1: 1000, Abcam), AMPK (Cat. No.: ab32047; 1: 1000, Abcam), p-AMPK (Cat. No.: ab133448; 1: 1000, Abcam), ULK1 (Cat. No.: ab128859; 1: 1000, Abcam), p-ULK1 (Cat. No.: ab133765; 1: 1000, Abcam), Beclin1 (Cat. No.: ab207612; 1: 1000, Abcam), LC3(Cat. No.: ab48394; 1: 1000, Abcam), and p62 (Cat. No.: ab109012; 1: 1000, Abcam), followed by incubating at 4 °C overnight. Afterwards, the mixture was incubated with the goat anti-rabbit horseradish peroxidase (HRP)-conjugated immunoglobulin G (IgG) secondary antibodies (No.: ab6721; 1: 10000, Abcam) at 37 °C for one hour, followed by visualization with the Leica LAS X Gel Documentation & Analysis System. The membranes were probed with the actin serving as the loading control. The semi-quantitative analysis of the bands was performed using the ImageJ software. 

### Establishment of the EMs mouse model

***This study was conducted at the Animal Experiment Centre of the Fourth Affiliated Hospital of Soochow University. All experimental procedures and animal management protocols were approved by the Institutional Animal Care and Use Committee (IACUC) of the Fourth Affiliated Hospital of Soochow University (Approval No. 251199).***

The sample size was determined based on a previous description (16) to ensure adequate statistical power while adhering to the principles of the 3Rs, particularly reduction. Sample size was calculated with the significance level (α) set at 0.05 and the desired statistical power (1-β) at 0.8. The calculation indicated that at least eight mice per group would be required to detect the expected biological effect. To account for potential experimental loss, we ultimately included 10 mice per group. A total of 30 female C57BL/6 mice (8 weeks old, weighing 18–22 g; strain number N000013; purchased from Gempharmatech) were utilized. The animals were housed in M1-type cages at a controlled room temperature of 20–25 °C, with 10 mice per cage, ensuring appropriate ventilation and ample space for movement. Animals had *ad libitum* access to standard pellet feed and water. Daily health status checks and environmental monitoring were performed; bedding was replaced 2–3 times per week, and environmental enrichment (e.g., paper nests and paper rolls) was provided to mitigate stress.

The estrous cycle was monitored by daily vaginal cytology for seven consecutive days prior to modeling. On the modeling day (8 weeks of age), 76.7% of the mice were in proestrus to estrus, and tissues were collected from all donor mice (n = 10) at this stage. The remaining 20 female mice were randomly divided into two groups. After a 7-day acclimatization period, 20 mice were randomly assigned as recipient mice, and 10 mice were designated as donor mice. Donor mice were euthanized by inhalation of an excessive dose of isoflurane anesthesia, with loss of the righting reflex and cessation of breathing used to confirm deep anesthesia prior to cervical dislocation for verification of death. The uteri were harvested, washed in sterile saline, and dissected longitudinally along the uterine horns into small fragments approximately 1 mm in diameter. The endometrial fragments were suspended in sterile saline at a donor tissue to recipient ratio of 1:2 and transplanted intraperitoneally into the recipient mice to induce an endometriosis-like model. All surgical procedures were performed under aseptic conditions to minimize tissue trauma.

Recipient mice were closely monitored for signs of postoperative pain. If pain-related behaviors (e.g., persistent curling, inappetence, wound scratching) were observed, subcutaneous administration of ibuprofen (10 mg/kg) was provided for analgesia as necessary.

### Animal grouping and experimental treatments

Each mouse was assigned a unique identification number, and a random number sequence was generated using a random number generator to allocate animals into groups. The 20 recipient mice were randomly assigned to two groups (n = 10 per group):

• **PTL group:** Received intraperitoneal injections of PTL (10 mg/kg) three times per week, commencing one hour before endometrial transplantation.

• **Sham group:** Received an equal volume of vehicle (1% DMSO in PBS; 40 µl) administered intraperitoneally three times per week.

The experimental duration was 4 weeks. At the conclusion of the study, all animals were deeply anesthetized via isoflurane inhalation to ensure complete loss of consciousness and absence of pain prior to tissue harvesting. Humane endpoints were defined as sustained weight loss exceeding 20%, severe wound infection, or overt signs of distress (e.g., hunched posture, labored breathing). No animals reached humane endpoints before the planned study endpoint. One mouse was dropped out of this study due to model failure. At the end of the experiment, all surviving animals were euthanized and were not reused for any additional studies. Only animals with histologically confirmed endometriosis-like lesions were included in the final data analysis, including hematoxylin-eosin (H&E) staining and immunohistochemistry (IHC).

### H&E staining

Tissue specimens were first fixed in 4% neutral buffered formalin for 24 h to ensure complete preservation of tissue morphology and cellular structure. The fixed specimens undergo dehydration with a gradient of ethanol concentrations (70%-100%), followed by clearing in xylene and final paraffin embedding. Paraffin-embedded tissue blocks are sectioned into 5μm-thick serial sections using a rotary microtome and mounted onto pretreated anti-shedding slides. Following routine dewaxing and rehydration, sections were stained using the H&E. Following staining, sections underwent dehydration, clearing, and neutral resin mounting. Histopathological changes, including tissue structural integrity and cellular morphological characteristics, were observed and evaluated under a Leica optical microscope.

### IHC

After paraffin removal with xylene and rehydration with graded ethanol, sections were placed in a 95 °C water bath for 20 min to perform heat-blocking in a pH 6.0 citrate buffer. Following cooling, sections were incubated at room temperature with 3% hydrogen peroxide for 10 min to block endogenous peroxidase activity. After washing with PBS, sections were blocked with 5% goat serum for 30 min. Then the sections were incubated with the specific primary antibodies, including GSDMD, IL-1β, caspase-1-p20, and NLRP3, followed by HRP-labeled secondary antibodies (Cat. No.: ab6721; 1:500, Abcam) to assess the inflammation- and pyroptosis-related protein expression. The positive and negative cells for GSDMD, IL-1β, caspase-1-p20, and NLRP3 in the cytoplasm was observed under a microscope to evaluate the proliferation in 5 randomly selected fields. The ImageJ software was used for the quantitative analysis, along with the Profiler, using the H-score by two experienced staff members blinded to this study design. 

### Statistical analysis

All the tests were performed at least in triplicate. All the data were presented as mean ± standard deviation. For data with normal distribution and homogeneity of variance, we applied standard ANOVA with appropriate post hoc multiple comparisons. For data that did not meet normality or homoscedasticity assumptions, we used non-parametric methods such as the Kruskal-Wallis test and Mann-Whitney U test. Data analysis was performed with the SPSS 26.0 software. A *P* value of less than 0.05 was considered to be statistically significant. 

## Results

### PTL down-regulated the expression of IL-1β and caspase-1-P20

**Figure 1:**
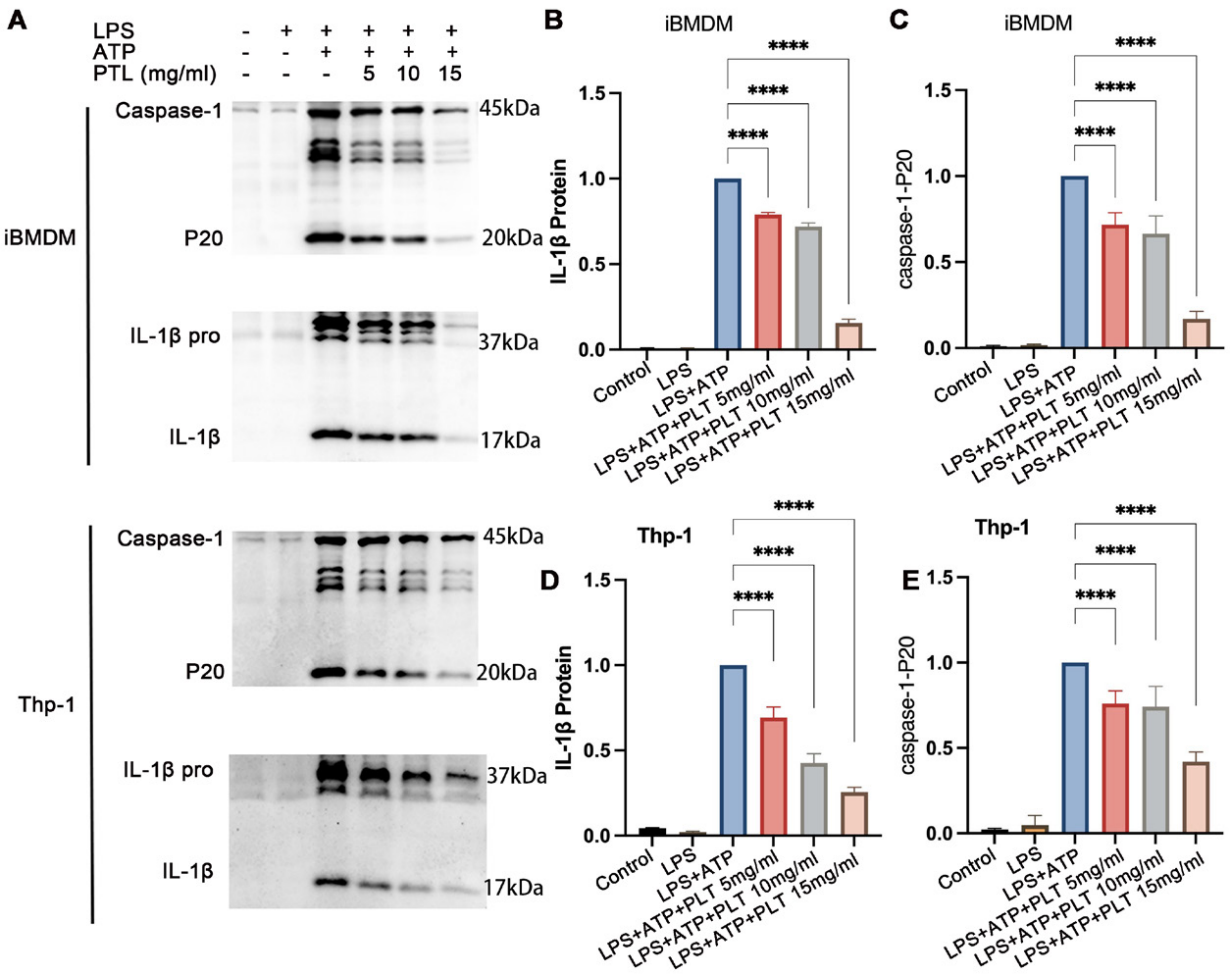
PTL down-regulated the expression of IL-1β and caspase-1-P20 in immortalized bone marrow-derived macrophages (iBMDM) and THP-1 cells

To evaluate the regulatory effect of PTL on NLRP3 inflammasome activation, we examined the expression levels of IL-1β and cleaved caspase-1-p20 in THP-1 and iBMDM cell models. In both cell types, IL-1β and caspase-1-p20 expression remained minimal under basal conditions (control group) and following LPS stimulation alone. However, upon combined LPS and ATP stimulation, a marked up-regulation of IL-1β and caspase-1-p20 was observed, indicating robust NLRP3 inflammasome activation. Notably, treatment with PTL significantly attenuated the expression of both IL-1β and caspase-1-p20 in a dose-dependent manner, particularly at higher PTL concentrations (all *P*<0.05, Figure 1A-1E). These findings suggest that PTL effectively inhibits NLRP3 inflammasome activation, thereby suppressing downstream proinflammatory signaling cascades. 

### PTL regulated the NLRP3/caspase-1/GSDMD-mediated pyroptosis via inhibiting the GSDMD lysis and ASC aggregation

**Figure 2:**
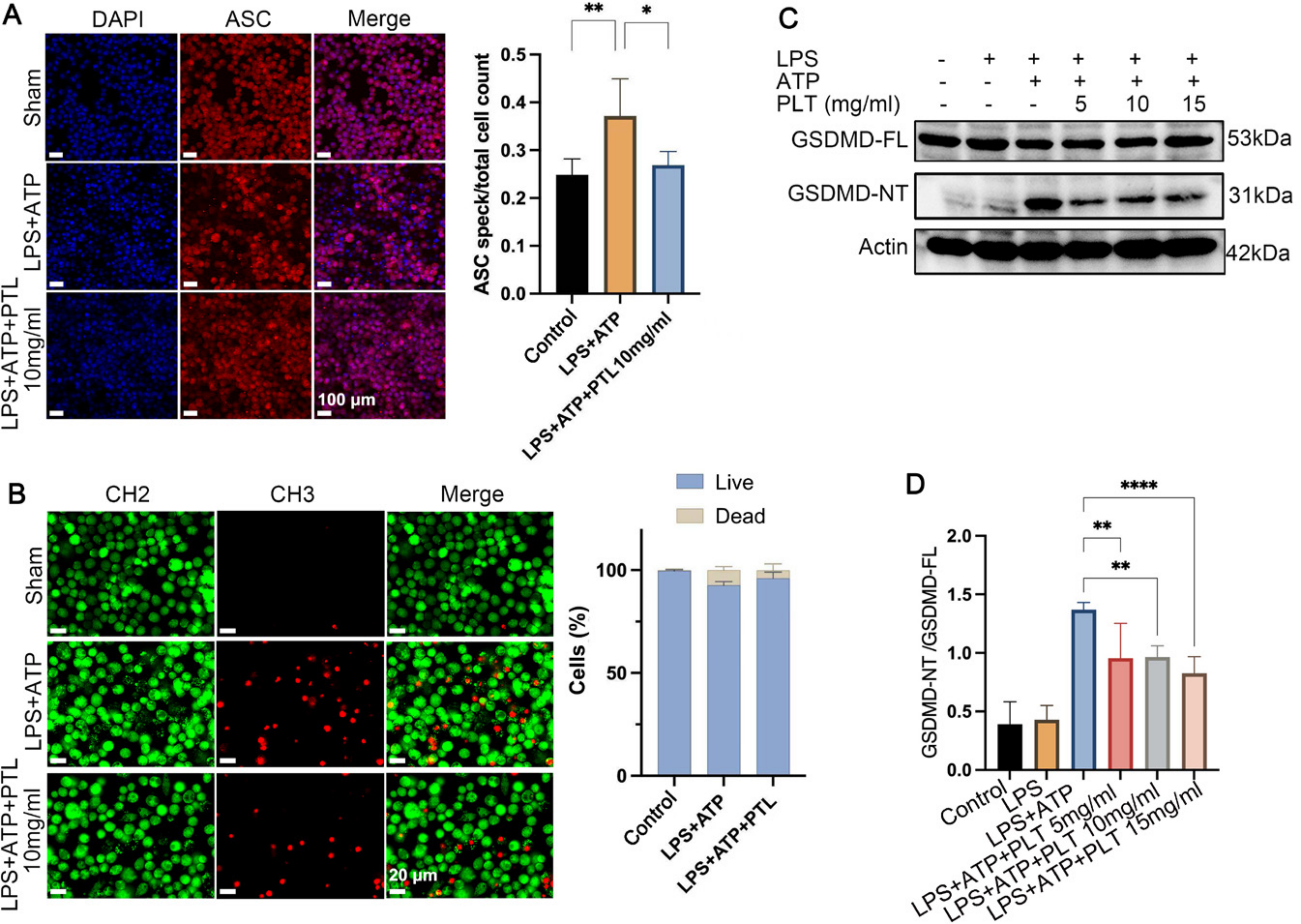
Roles of PTL in modulating the pyroptosis and the potential mechanisms

ASC speck formation is a key step in the activation of inflammasomes, and is an early event in inflammatory response and pyroptosis. Immunofluorescence staining results showed that no obvious ASC speck formation was observed in the cells of the control group, and the ASC protein was diffusely distributed with almost no aggregation (Figure 2A). In the LPS+ATP group, obvious bright ASC speck punctate aggregation was observed in the cells, and the number of ASC specks in the LPS+ATP+PTL (10 mg/ml) group was significantly reduced compared with the LPS+ATP group. The primary cellular death was pyroptosis in the LPS+ATP group, and the pyroptosis was significantly inhibited in the presence of PTL** (**Figure 2B**). **To investigate the regulatory roles of PTL in NLRP3/caspase-1/GSDMD-mediated pyroptosis, we determined the expression of N-terminal poreforming GSDMD fragment (GSMDM-NT) and full-length GSDMD (GSDMD-FL). As shown in Figures 2C and 2D, compared with the control and LPS groups, the GSDMD-NT/GSDMD-FL ratio in the LPS+ATP groups showed a significant increase (all *P*<0.001), which indicated that PTL treatment inhibited the cleavage of GSDMD. Besides, the expression of GSDMD showed a significant decrease in the LPS+ATP+PTL groups, in a PTL-dose-dependent manner. This indicated that PTL inhibited the pyroptosis through inhibiting the activation of the NLRP3/caspase-1/GSDMD pathway. 

### PTL induced down-regulation of NLRP3 and pro-IL-1β

**Figure 3:**
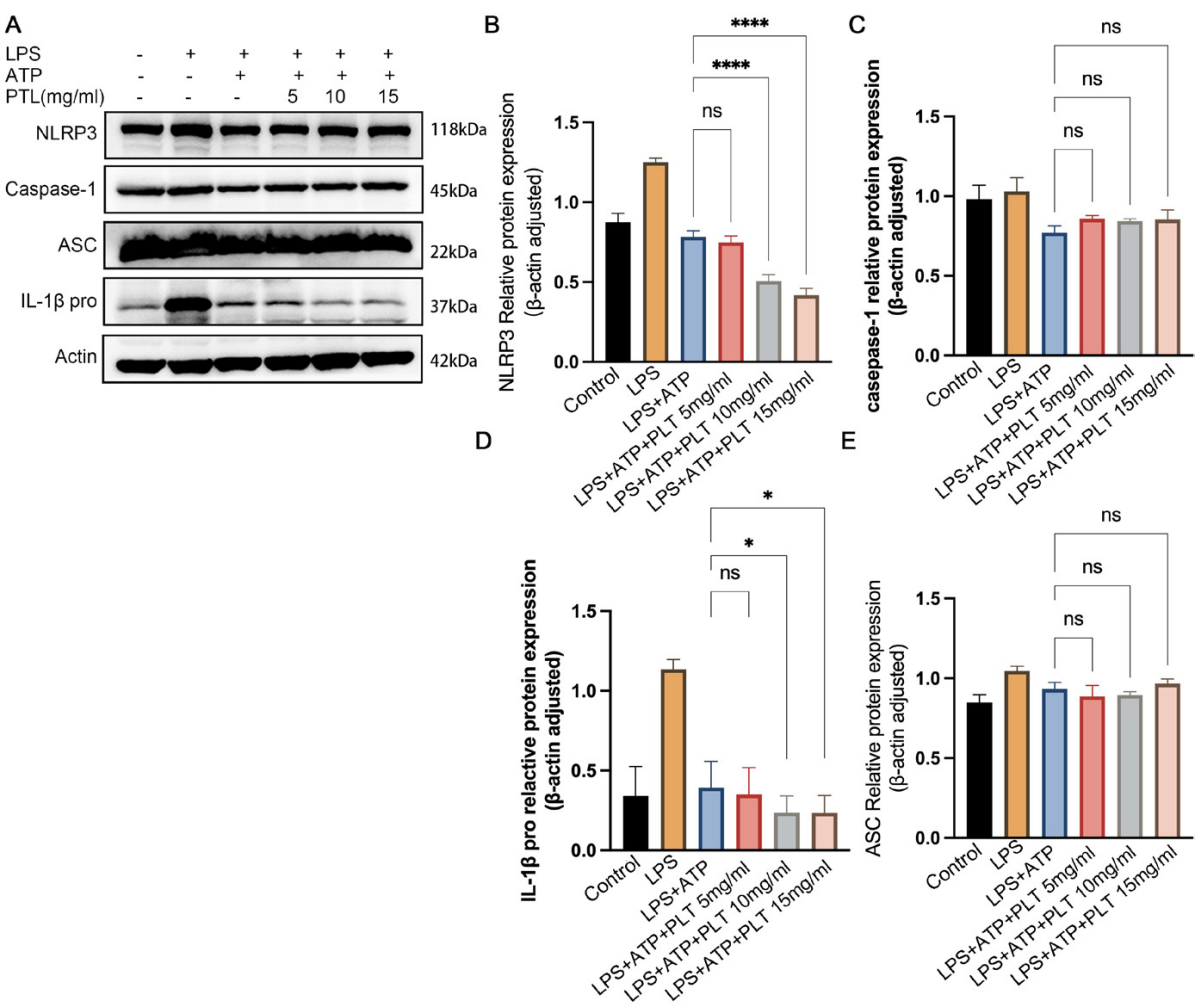
Western blot analysis revealed the effects of different concentrations of PTL on the NLRP3 inflammasome pathway (NLRP3, ASC, IL-1β, caspase-1-P20)

PTL treatment significantly reduced the expression of NLRP3 (Figures 3A and B) and pro-IL-1β (Figures 3A and D) in a dose-dependent manner, suggesting an inhibitory effect on inflammasome priming and cytokine precursor synthesis. In contrast, the protein levels of ASC and caspase-1 were not significantly altered (all *P*>0.05, Figures 3C and E), indicating that PTL may not directly affect these inflammasome components at the expression level.

### PTL modulated the IL-1β and caspase-1-p20 expression by affecting the autophagy

**Figure 4:**
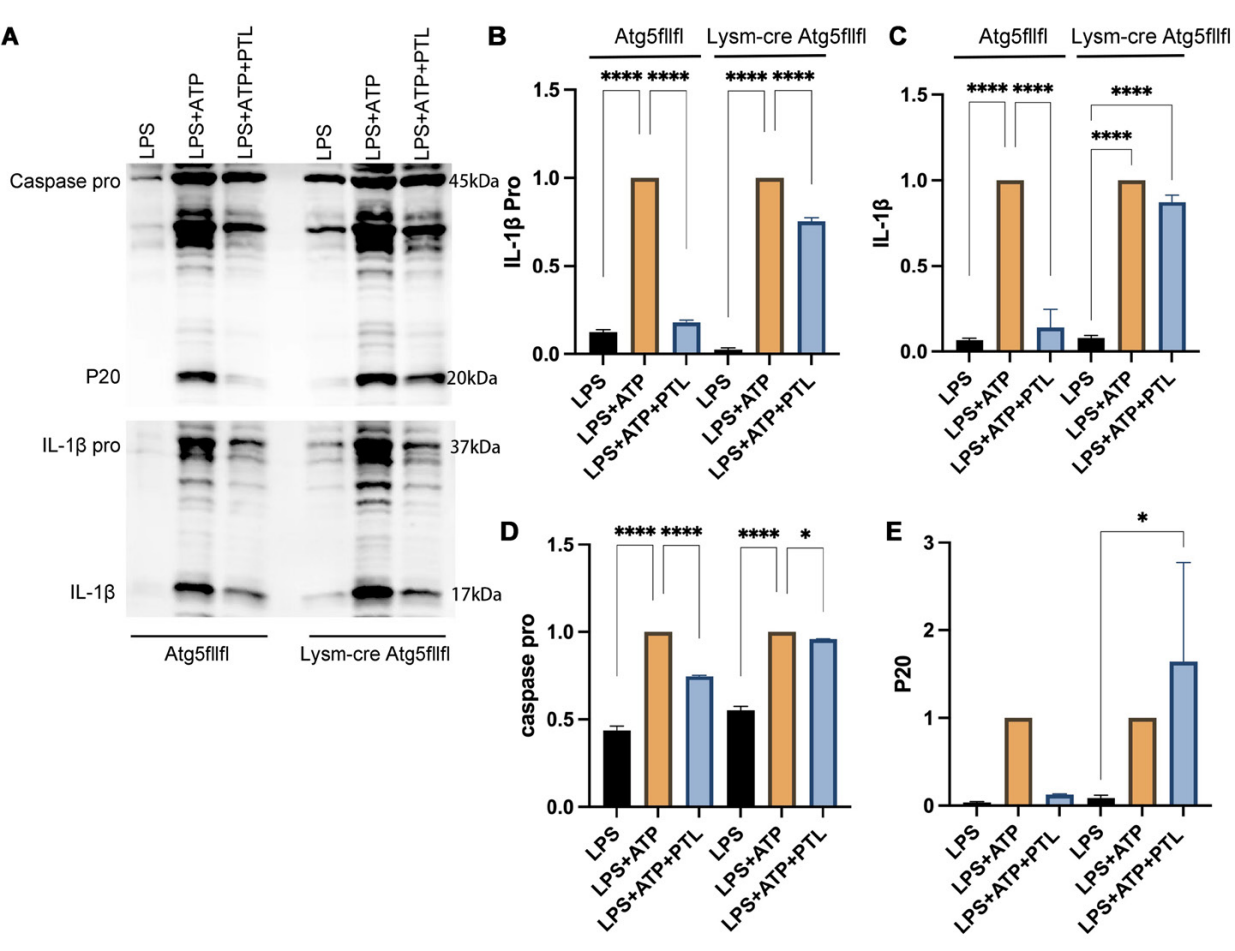
PTL modulated the IL-1β and Caspase-1-p20 expression via affecting the autophagy

**Figure 5:**
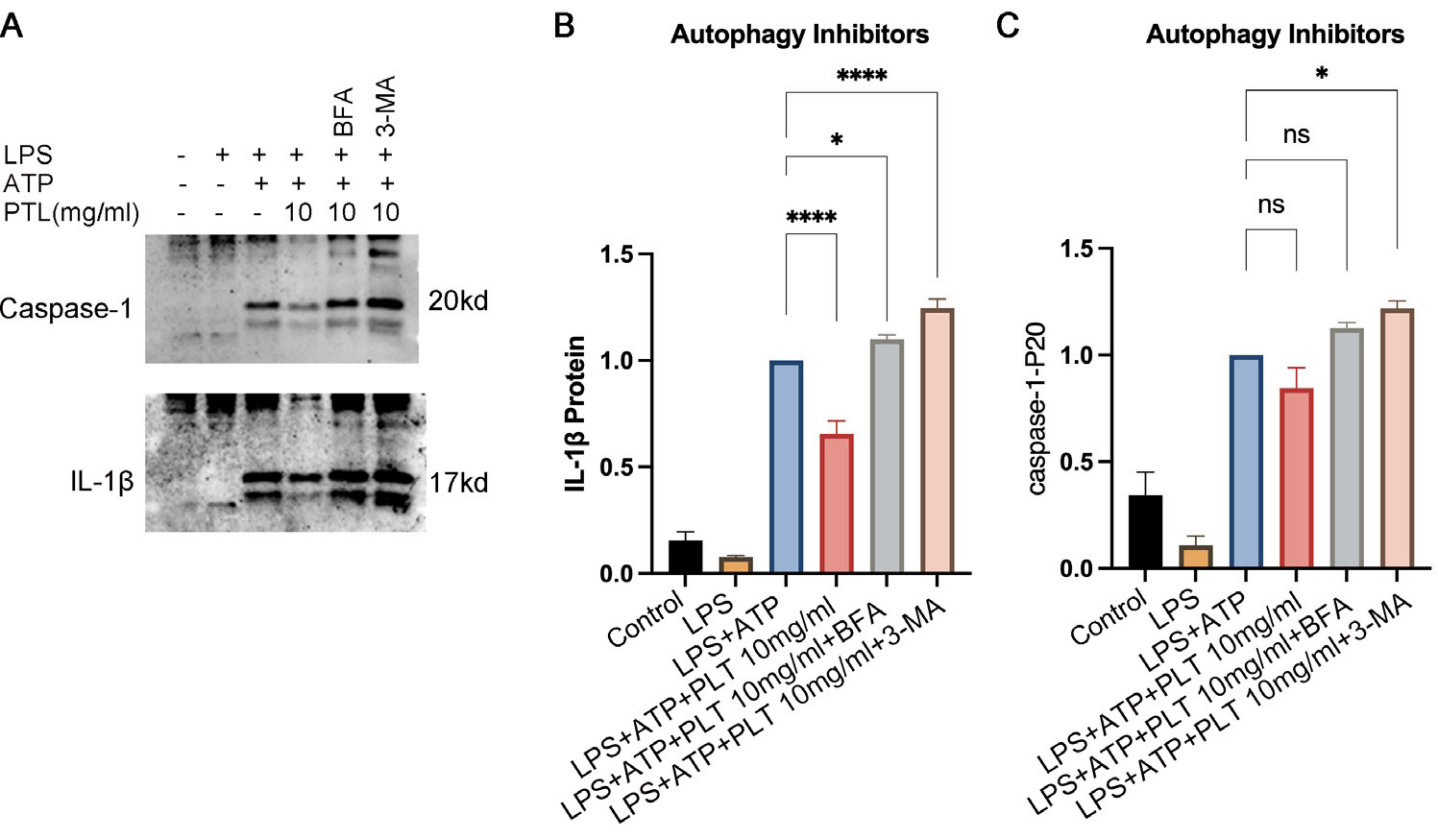
Effect of PTL on the NLRP3 inflammatory vesicle pathway (IL-1β, caspase-1-P20) in the presence of the autophagy inhibitors BFA and 3-MA

In the presence of autophagy (Atg5fl/fl group), the expression of IL-1β and caspase-1-p20 was significantly up-regulated in the LPS+ATP group (*P*<0.001), and was significantly down-regulated by PTL (*P*<0.001) (Figure 4A-E). In contrast, in the absence of autophagy after knocking down the Atg5fl/fl driven by Lysm-cre, there were no statistical differences in the expression of IL-1β and caspase-1-p20 in the LPS+ATP+PTL group compared with the LPS+ATP group (all *P*>0.05). To further illustrate the roles of autophagy in the inflammatory pathways, we determined the expression of IL-1β and Caspase1-p20 in the presence of autophagy inhibitors (i.e., 3-MA and BFA). In the presence of an autophagy inhibitor, the down-regulation of IL-1β and caspase-1-p20 mediated by PTL was no longer available, which was featured by no statistical differences in the IL-1β and caspase-1-p20 expression in the LPS+ATP+PTL group compared with the LPS+ATP group (Figures 5A and 5B). This indicated that the PTL inhibited NLRP3 inflammasome activation via regulating autophagy. 

### PTL modulated the expression of autophagy-related protein and the activation of the AMPK/ULK1 signaling pathway

**Figure 6:**
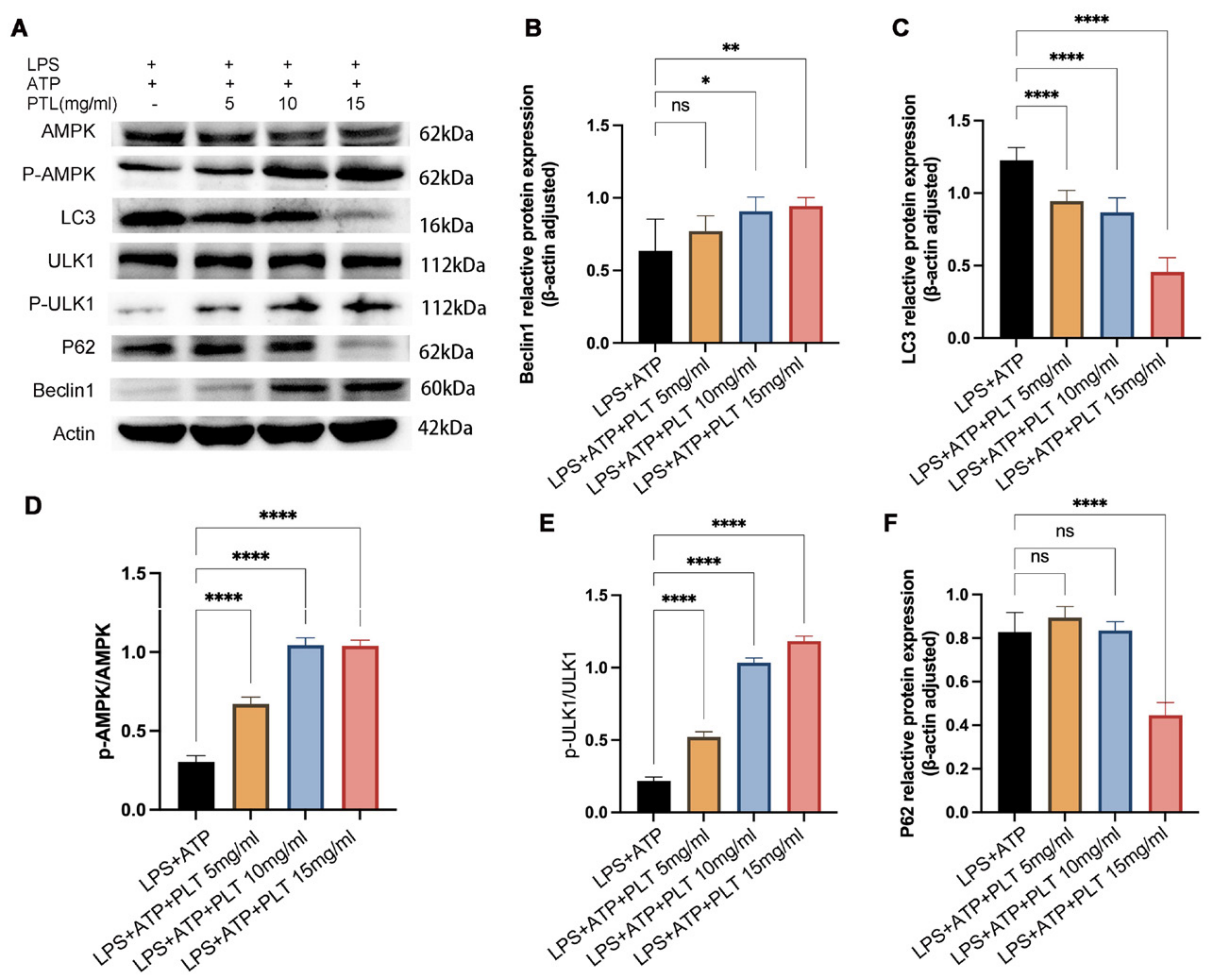
The regulatory effect of PTL on the expression levels of autophagy-related proteins (Beclin1, LC3, AMPK, ULK1, p-AMPK, p-ULK1, p62) under LPS+ATP stimulation conditions

In this section, we determined the expression of autophagy-related protein (e.g., Beclin1 and LC3) and the activation of the AMPK/ULK1 signaling pathway (Figure 6A). PTL (10 mg/ml and 15 mg/ml) induced significant up-regulation of Beclin1 in a dose-dependent manner compared with the LPS+ATP group (all *P*<0.05, Figure 6B). The expression of LC3 was significantly down-regulated in the LPS+ATP+PTL group compared with the LPS+ATP group, in a dose-dependent manner (all *P*<0.05, Figure 6C). The expression of p-AMPK and p-ULK1 protein was significantly up-regulated in the LPS+ATP+PTL group compared with the LPS+ATP group, presenting in a dose-dependent manner (all *P*<0.05, Figures 6D and E). PTL (15 mg/ml) triggered significant down-regulation of p62 compared with the LPS+ATP group (*P*<0.0, Figure 6F), while no statistical differences were noticed between the expression of p62 in the PTL with a concentration of 5 mg/ml and 10 mg/ml compared with the LPS+ATP group. All these indicated that the PTL contributed to the autophagy process. 

### Effects of PTL on EMs-like lesions in mice

**Figure 7:**
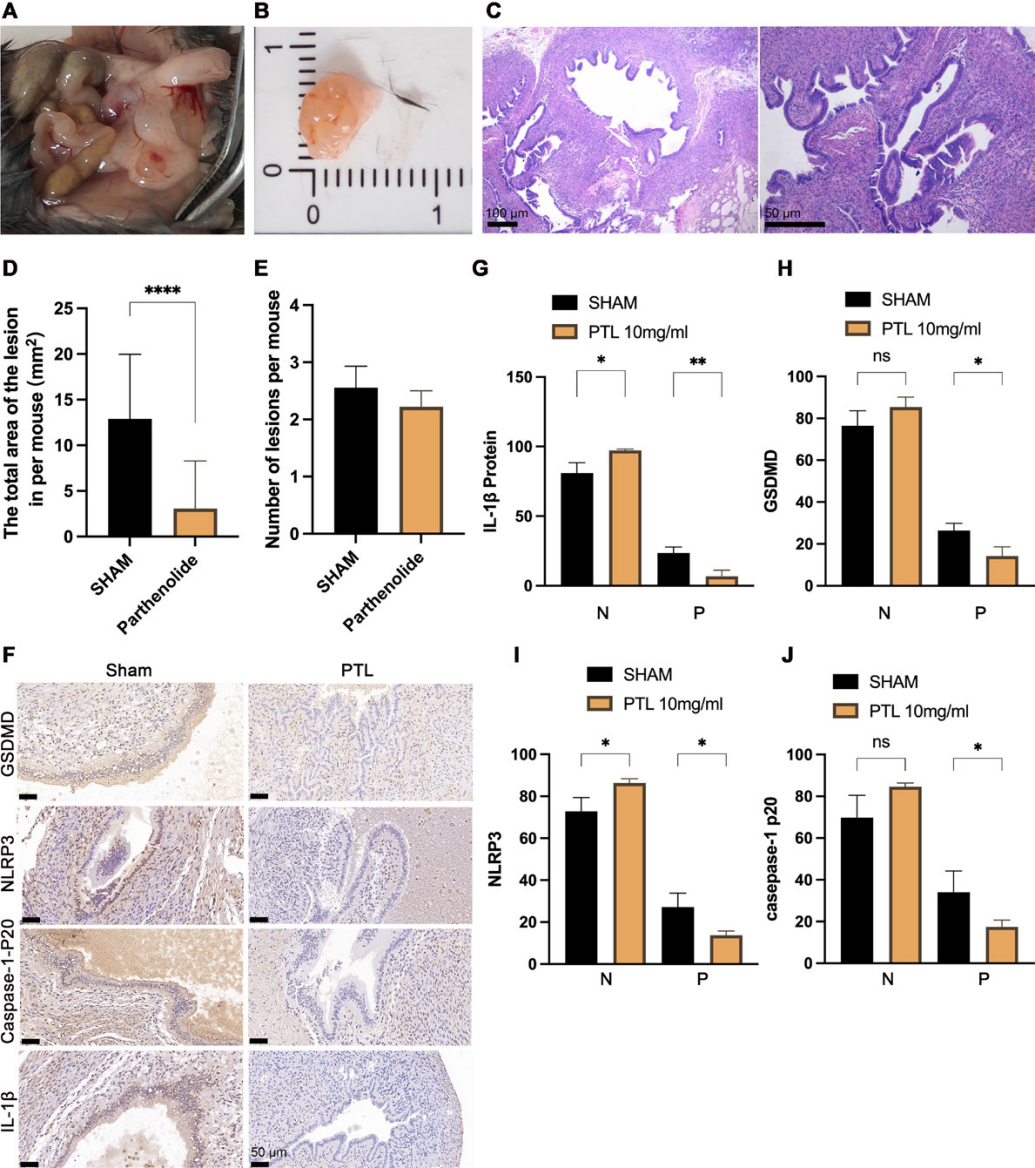
Effects of PTL on EMs-like lesions in mice

Both the sham group (n=10) and the PTL group (n=10) were included in the analysis based on pathological characteristics. The majority of the lesions were localized to the injection site, peritoneum, and the site adjacent to the uterus, with a lesion size of approximately 2-6 mm (Figures 7A and B). The HE staining results for the animals in the sham and PTL groups are shown in Figure 7C. About 4 weeks after intraperitoneal injection of PTL, the local area of lesions per mouse in the PTL group showed a significant decrease compared with that of the sham group (*P*<0.001, Figure 7E), but the number of lesions showed no difference (2.22±0.28 vs. 2.56±3.78, *P*>0.05, Figure 7F). 

Besides, we also determined the proportion of NLRP3, GSDMD, caspase-1-p20, and IL-1 expressing endometrial epithelial cells, which showed a statistical decrease in the PTL group compared with the sham group (NLRP3: 13.74% vs. 27.22%, *P*<0.05; GSDMD: 14.20% vs. 26.41%, *P*<0.05; caspase-1-p20: 17.49% vs. 34.00%, *P*<0.05; IL-1β: 6.51% vs. 23.49%; Figure 7D, 7G-J).

## Discussion

The imbalance between inflammation and autophagic function plays a pivotal role in the pathogenesis of EMs (17). Activation of inflammatory pathways, such as the NLRP3 inflammasome, has been shown to inhibit autophagy through multiple mechanisms (18). Conversely, impaired autophagy hinders the clearance of pathogenic factors under sustained inflammatory conditions, further contributing to lesion progression. As a naturally occurring sesquiterpene lactone, PTL has been shown to possess multiple biological activities, including anti-inflammatory and anti-oxidant effects. However, its therapeutic potential and underlying mechanisms in the context of EMs remain inadequately explored. 

In this study, we demonstrated PTL functions as a dual modulator, enhancing autophagy while suppressing NLRP3 inflammasome activation under LPS and ATP-induced inflammatory conditions. *In vitro*, PTL markedly reduced the expression of NLRP3, cleaved caspase-1-p20, and IL-1β, and attenuated pyroptosis via down-regulation of GSDMD. Concomitantly, PTL enhanced autophagic activity, as indicated by increased Beclin1 levels, enhanced phosphorylation of AMPK and ULK1, and reduced p62 and LC3 accumulation, suggesting improved autophagic flux. *In vivo*, PTL treatment alleviated inflammatory responses in EMs-like lesions, evidenced by diminished inflammasome activation and decreased secretion of IL-1β, along with down-regulation of GSDMD. The formation of ASC speck is a direct manifestation of ASC protein oligomerization during the inflammasome assembly process and is a key link in the inflammatory response and cell pyroptosis. In this study, the addition of PTL effectively inhibited the production of ASC speck, indicating that PTL can block the inflammasome activation process induced by LPS+ATP and has a negative regulatory effect. Notably, PTL administration significantly reduced the number, size, and weight of ectopic lesions. These findings reveal a previously unrecognized mechanism by which PTL interrupts inflammatory-autophagy crosstalk and highlight its potential as a non-hormonal therapeutic strategy for EMs.

PTL has emerged as a promising model compound for the development of novel anti-leukemia and anti-tumor therapies (19). As a thiol-reactive electrophilic molecule, it has been shown to modulate multiple critical cellular signaling pathways, many of which are implicated in the pathogenesis of cancer and degenerative disorders (20). Notably, recent findings in the context of EMs highlight the regulatory effects of PTL on the NLRP3/IL-1β/caspase-1-p20 inflammatory axis, underscoring its therapeutic potential in mitigating disease-associated inflammatory responses. By selectively targeting this pathway, PTL may effectively suppress the localized inflammatory microenvironment characteristic of endometriotic lesions, thereby offering a mechanistically distinct alternative to conventional hormone-based therapies. Importantly, in contrast to the well-documented adverse effects of hormonal treatments, herbal compounds such as PTL exhibit favorable safety profiles and are well tolerated, owing to their indirect modulation of the endocrine system. These properties suggest PTL may represent a promising strategy for the long-term management of EMs with minimal side effects.

Although PTL has shown significant anti-inflammatory effects and the potential to promote autophagy in EM models, its clinical application as a new therapeutic agent still faces many challenges. First, the low oral bioavailability of PTL may limit its ability to reach effective therapeutic concentrations *in vivo*. Currently, most studies are still in the *in vitro *or animal experimental stage, lacking validation of their efficacy and safety in humans. Although a phase I dose-escalation clinical trial for cancer patients showed no obvious adverse reactions after daily oral administration of 4 mg PTL, pharmacokinetic parameters could not be obtained because no quantifiable concentration was detected in plasma (21). This indicates that it is still necessary to improve its purity and dosage form to achieve higher doses. Second, although studies have shown that PTL can inhibit key inflammatory factors such as IL-1β, its specific regulated signaling pathways and its synergistic mechanism with other inflammatory mediators are still unclear. In a complex physiological and pathological environment, individual differences in patients and heterogeneity of lesions may affect the therapeutic effect. In addition, autophagy is a highly dynamic cellular process, and its overactivation may induce cell death or dysfunction, and may even aggravate tissue damage in certain pathological situations. This also brings uncertainty to the therapeutic application of PTL. Therefore, systematic preclinical and clinical studies are still needed to clarify further its safety, efficacy, and potential side effects, and provide a solid basis for its clinical transformation in the treatment of EMs.

The NLRP3/caspase-1/IL-1β-mediated pyroptosis pathway plays a pivotal role in the pathogenesis of EMs (22). Upon activation, the NLRP3 inflammasome recruits the adaptor protein ASC, which in turn facilitates the activation of procaspase-1. Active caspase-1 subsequently cleaves the precursor forms of IL-1β and IL-18 into their biologically active mature forms, while also cleaving GSDMD. The released N-terminal fragment of GSDMD translocates to and integrates into the plasma membrane, disrupting membrane integrity by altering transmembrane ion flux and ultimately inducing pyroptosis. During this process, cellular contents, including mature IL-1β and IL-18, are released into the extracellular space, thereby amplifying the local inflammatory response (23). Key components of this pathway, including NLRP3, caspase-1, pro-IL-1β, and IL-1β, are thus essential mediators of pyroptosis, and elevated secretion of IL-1β and IL-18 serves as a hallmark of this inflammatory form of cell death. Consistent with this, Kato et al. (24) demonstrated the presence of IL-1β in both ascitic fluid and serum samples from patients with EMs, and found that NLRP3 inflammasome activation in murine models was closely associated with lesion progression. These findings support the notion that therapeutic strategies targeting the NLRP3 inflammasome signaling axis may hold promise for mitigating disease severity in EMs. The retrograde menstruation theory remains the most widely accepted explanation for the origin of EMs, proposing that viable endometrial cells reflux into the peritoneal cavity during menstruation and subsequently implant ectopically. The mouse model employed in the present study was established based on this hypothesis. Prior studies have reported that PTL, a natural sesquiterpene lactone, suppresses both mitotic activity and inflammatory signaling in endometriotic lesions (16). Our current findings further substantiate these observations, demonstrating that PTL effectively attenuates lesion-associated inflammation and may serve as a viable therapeutic agent for the treatment of EMs.

Autophagy is a highly conserved cellular degradation pathway that maintains intracellular homeostasis and viability by removing damaged organelles and misfolded or aggregated macromolecules (25). It plays a central role in the pathogenesis of various cancers and tumor-related disorders. Core autophagy-related proteins, including Atg3, Atg5, Atg7, and Atg13, together with additional regulatory factors, are crucial in restraining tumor development (26). These proteins orchestrate autophagosome membrane initiation, elongation, and fusion, thereby modulating cell proliferation, survival, and metabolic adaptation. In the present study, we found that PTL markedly enhanced autophagy initiation, which was associated with increased phosphorylation of AMPK and ULK1. Up-regulation of Beclin1, restoration of LC3 processing, and a significant reduction in p62 accumulation indicated that PTL effectively activated autophagy and rescued the autophagic flux impaired by LPS+ATP stimulation. These findings, consistent with previous reports (27), suggest that PTL promotes the clearance of inflammation-associated cellular debris through autophagic pathways, contributing to cytoprotective and anti-inflammatory effects. Notably, autophagy has been identified as a negative regulator of NLRP3 inflammasome activation, attenuating inflammatory signaling through enhanced NLRP3 degradation and suppression of its phosphorylation (28, 29). Therefore, PTL-mediated autophagy activation may represent a critical mechanism underlying its anti-inflammatory action and inhibition of NLRP3 inflammasome activity.

(A-C) Changes in IL-1β and caspase-1-P20 protein levels in iBMDM in each group. (D,E) Expression of the IL-1β and caspase-1-P20 proteins in THP-1 (human monocytic leukaemia cell line). ****P<0.0001. PTL: Parthenolide

(A) ASC speck formation in each group. (B) The primary cellular death observed in the LPS+ATP group was confirmed to be pyroptosis, and the pyroptosis was significantly inhibited in the presence of PTL. (C, D) Cleavage of GSDMD-NT was significantly enhanced by LPS and ATP stimulation, whereas PTL treatment effectively reduced the generation of GSDMD-NT. **P<0.01; ****P<0.0001. PTL: Parthenolide

(A) Western blot results. (B) Semiquantitative analysis for the proteins. The bar graphs represent the mean ±standard deviation. *P<0.05; ****P<0.0001; ns, non-significant. PTL: Parthenolide

(A-E) IL-1β, IL-1β pro, caspase pro, and p20 protein levels in the Atg5fl/fl group were significantly elevated after LPS and ATP treatments, indicating the expression of inflammation, and the expression was significantly decreased by the addition of PTL. The inhibitory effect of PTL was significantly attenuated in the presence of ATG5 knock-down. *P<0.05; ***P< 0.001; ****P< 0.0001. PTL: Parthenolide

(A) Western blot analysis for the expression of IL-1β, caspase-1-P20. (B, C) Semiquantitative analysis for IL-1β, caspase-1-P20 in the presence of autophagy inhibitors. *P<0.05; ****P<0.0001; ns, non-significant. PTL: Parthenolide

(A) Western blot analysis for the expression levels of autophagy-related proteins. (B) PTL treatment significantly up-regulated the expression level of Beclin1. (C) LC3 expression gradually decreased with the increase of PTL concentration, suggesting that PTL may regulate autophagic flux. (D, E) The p-AMPK/AMPK and p-ULK1/ULK1 were significantly higher at higher PTL concentrations, suggesting that PTL acts as a facilitator of autophagosome initiation and progression through. (F) PTL promoted the degradation of p62 at high concentrations, thereby enhancing autophagic activity. *P<0.05; **P<0.01; ****P<0.0001; ns, non-significant. PTL: Parthenolide

(A) EMs-like lesions appearing in the abdominal cavity of mice. (B) Representative of a large excised lesion. (C) Hematoxylin and eosin staining of lesions under a magnification of 50× and 200×. (D, E) Comparison of surface area (E) and number (F) of abdominal endometriotic lesions in two groups of mice. (F) Immunohistochemical assay to detect the proportion of NLRP3, P20, GSDMD, and IL-1β expression in endometrioid lesions in mice under a magnification of 100×. (G-J). The semiquantitative analysis results for the IL-1β (G), GSDMD (H), NLRP3 (I), and caspase P20 (J). N: Negative or weakly expressed; P: Moderately to strongly expressed. *P<0.05; **P<0.01; ****P<0.0001; ns, non-significant. PTL: Parthenolide

## Conclusion

PTL was shown to simultaneously activate autophagy and suppress NLRP3 inflammasome activity, suggesting that it may alleviate local inflammation, inhibit fibrosis, and restore tissue homeostasis by disrupting this pathological cycle. As a potential therapeutic agent with dual regulatory properties, PTL offers a novel theoretical framework for treating EMs by modulating autophagy and NLRP3 inflammasome signaling pathways. It holds promise as an adjunct and maintenance strategy alongside hormonal or surgical treatments, with the potential to reduce recurrence and side effects. However, several key challenges remain for future research: determining the optimal administration route and dosage of PTL to maximize therapeutic efficacy while minimizing toxicity; developing more precise drug-delivery systems to enhance targeting efficiency; and designing rigorous clinical trials to validate its efficacy across different subtypes of EMs. 

## Data Availability

The data sets used and/or analyzed during the current study are available from the corresponding author upon reasonable request.

## Declaration

We have not used any AI tools or technologies to prepare this manuscript.
