# Supplementary material for: Parthenolide attenuated the endometriosis-like lesions by activating autophagy and suppressing NLRP3 inflammasome activity
Source: Iran J Basic Med Sci. 2026;29(1):90–100. doi: 10.22038/ijbms.2025.90575.19521 (PMC12867106; doi:10.22038/ijbms.2025.90575.19521)

Figure 1.A Example of original western blot

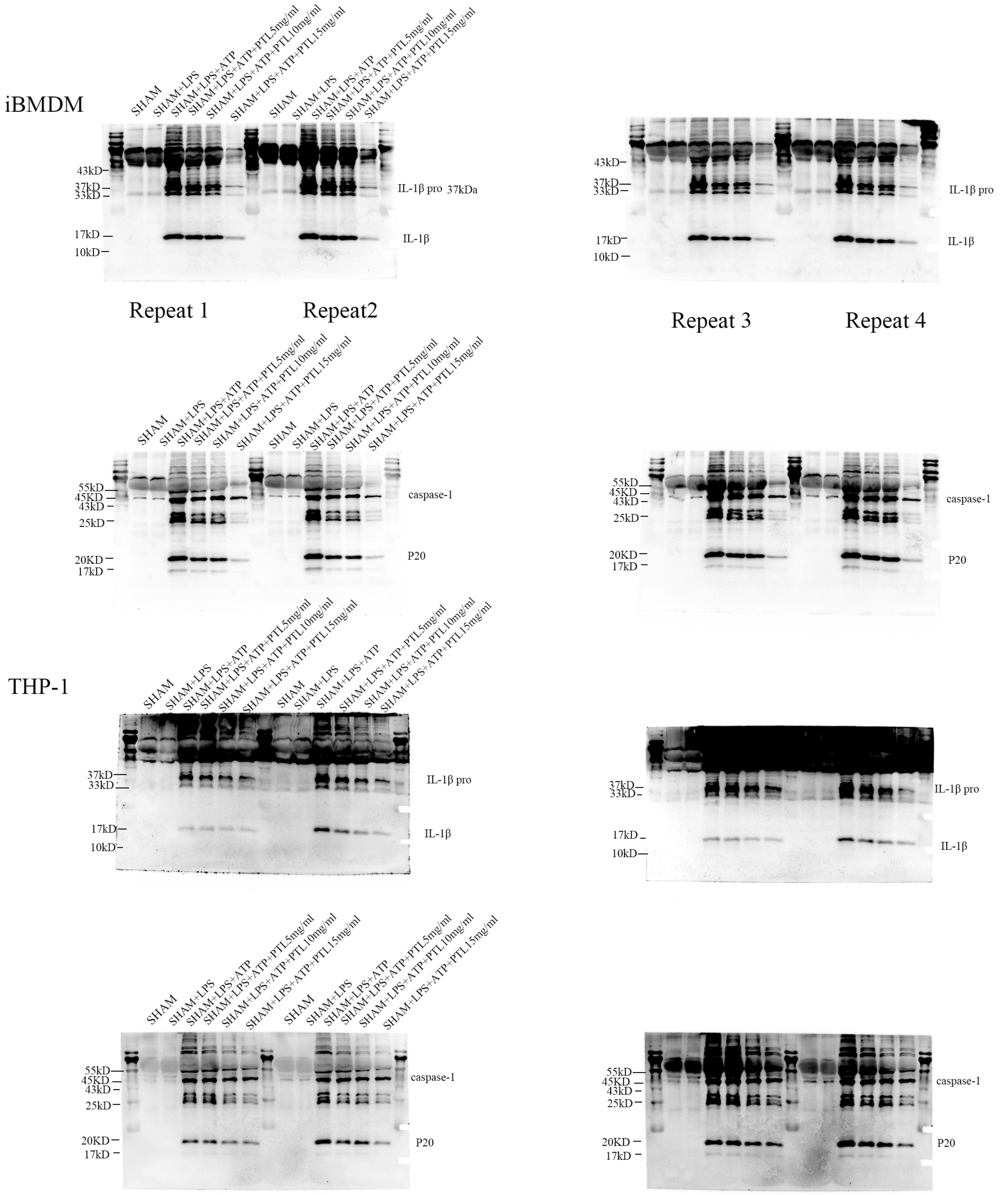

Figure 2. CExample of original western blot

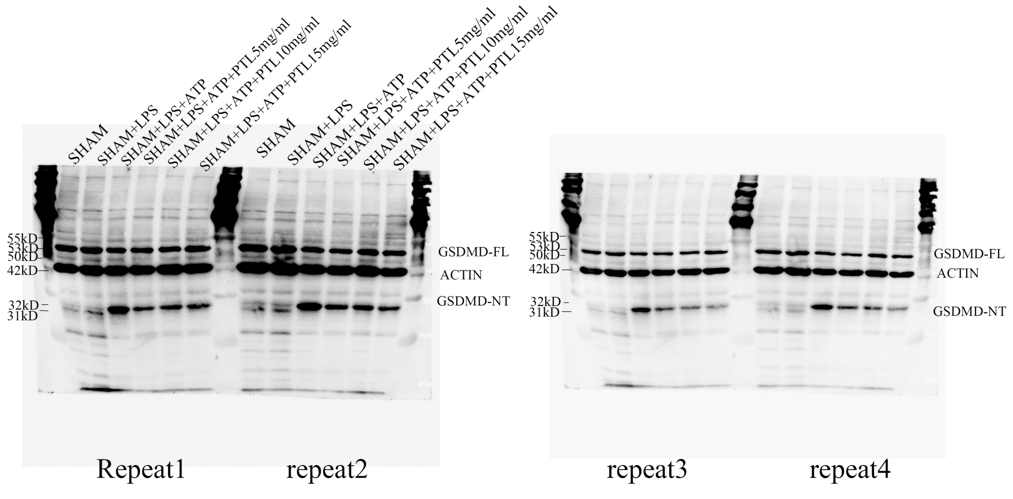

Figure 3.AExample of original western blot

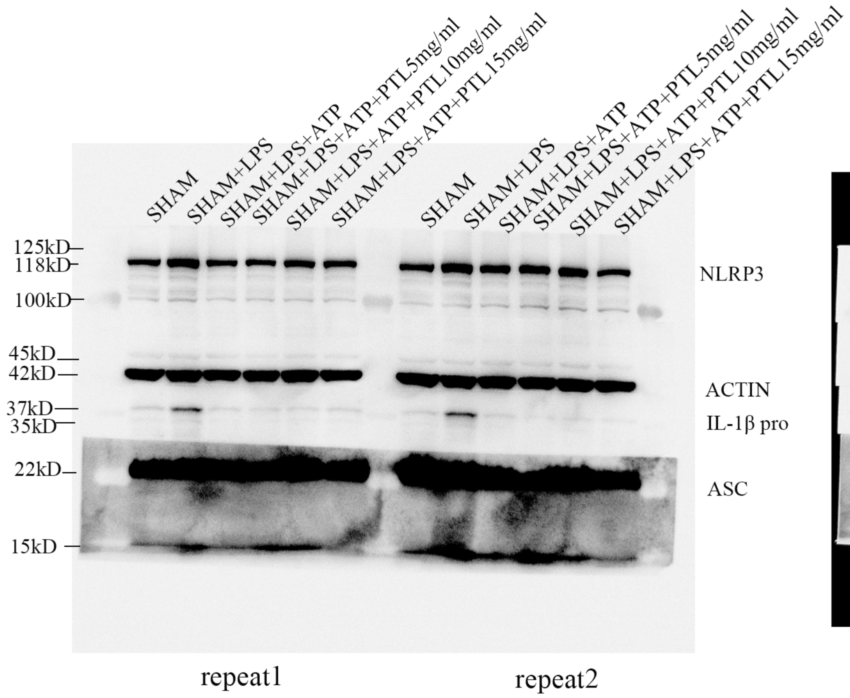

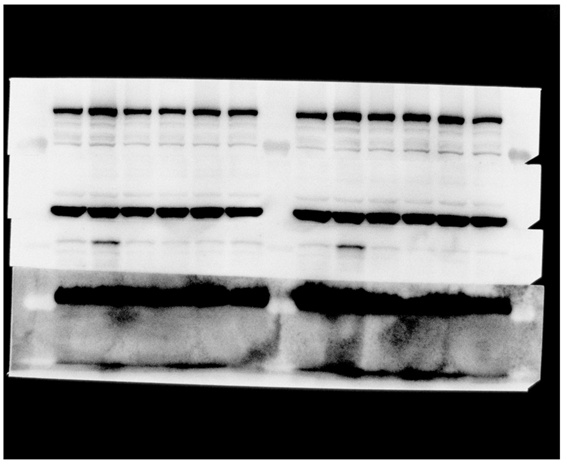

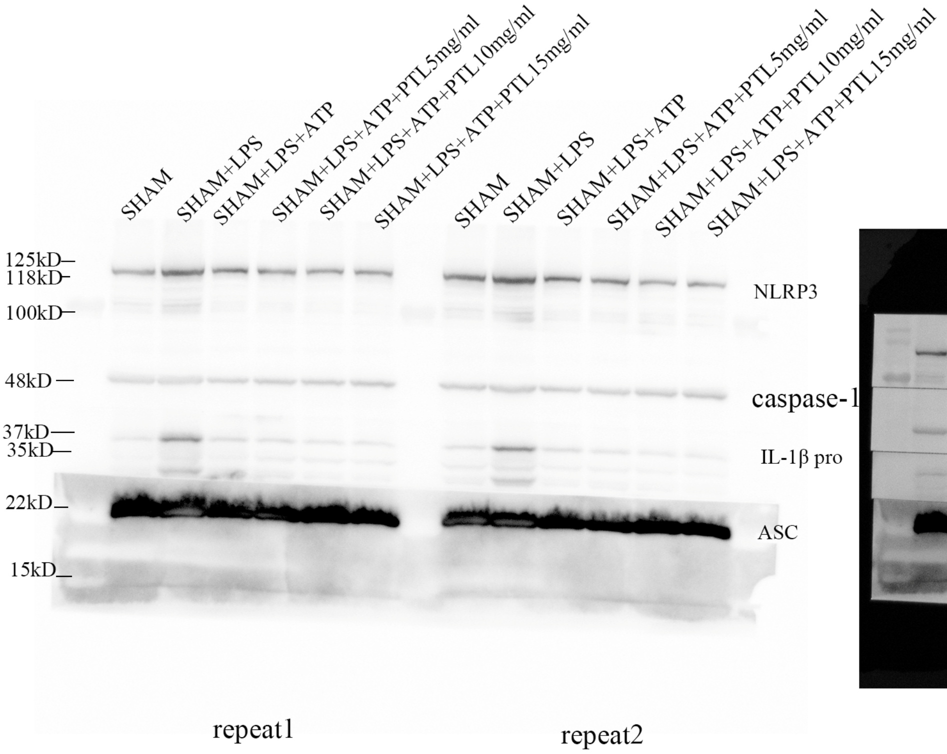

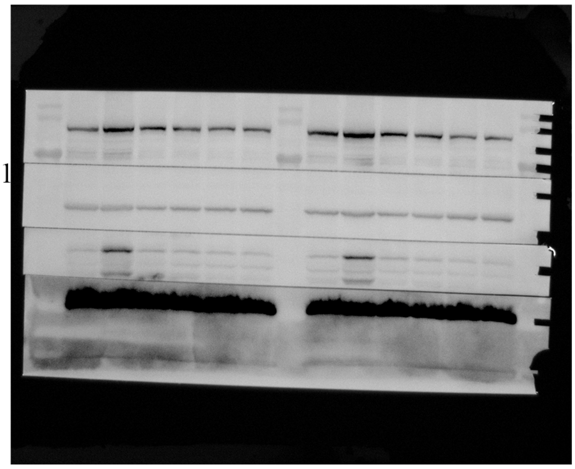

We used the same PVDF membrane.

In order to clearly show the individual protein bands in the thesis images,

we cropped the image of the corresponding region of each protein and arranged them in standard molecular weight order.

Figure 4.AExample of original western blot

The cell supernatant samples inherently lack endogenous reference proteins, justifying the absence of internal reference bands in the raw Western blot data.

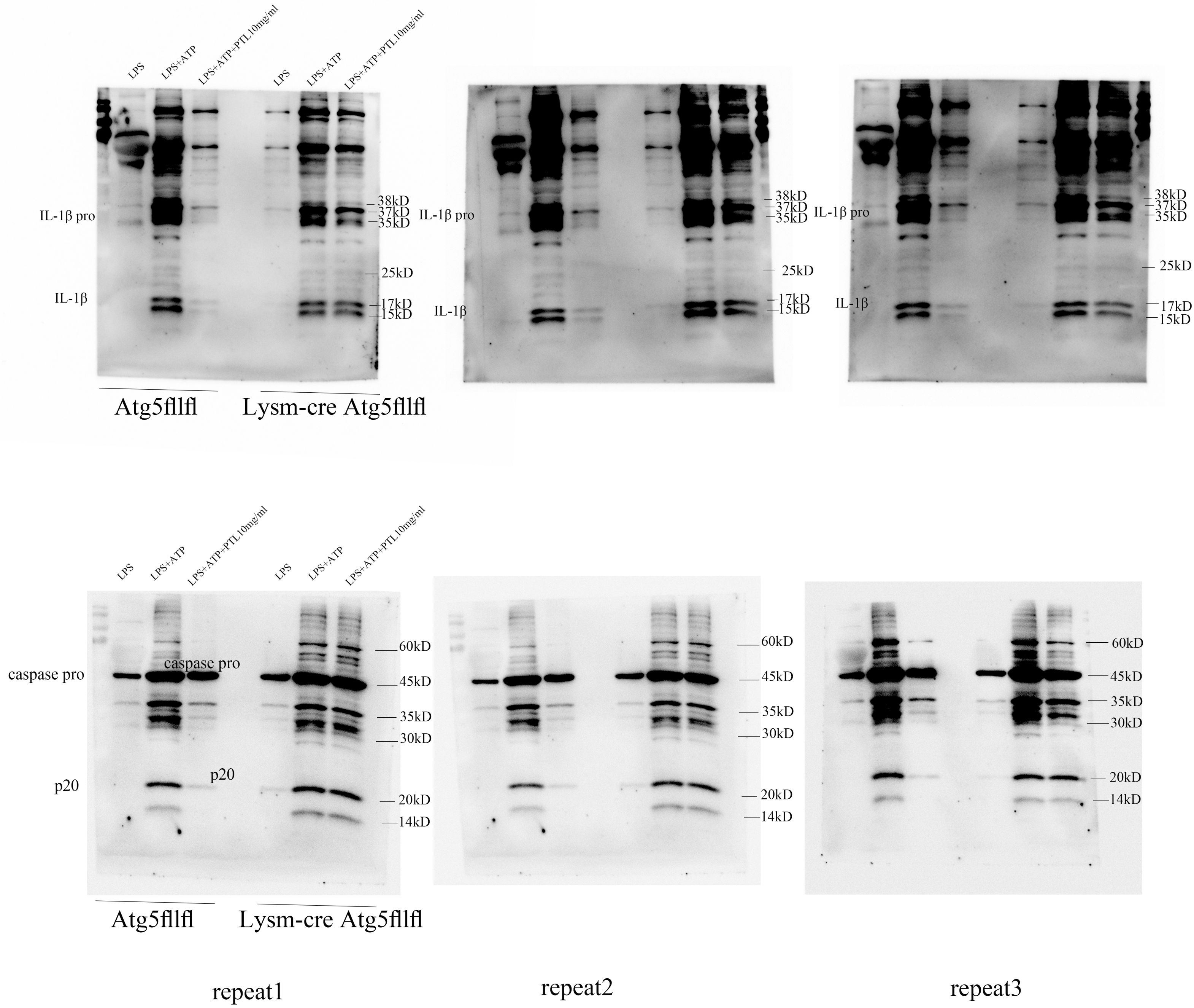

Figure 5.AExample of original western blot

The cell supernatant samples inherently lack endogenous reference proteins, justifying the absence of internal reference bands in the raw Western blot data.

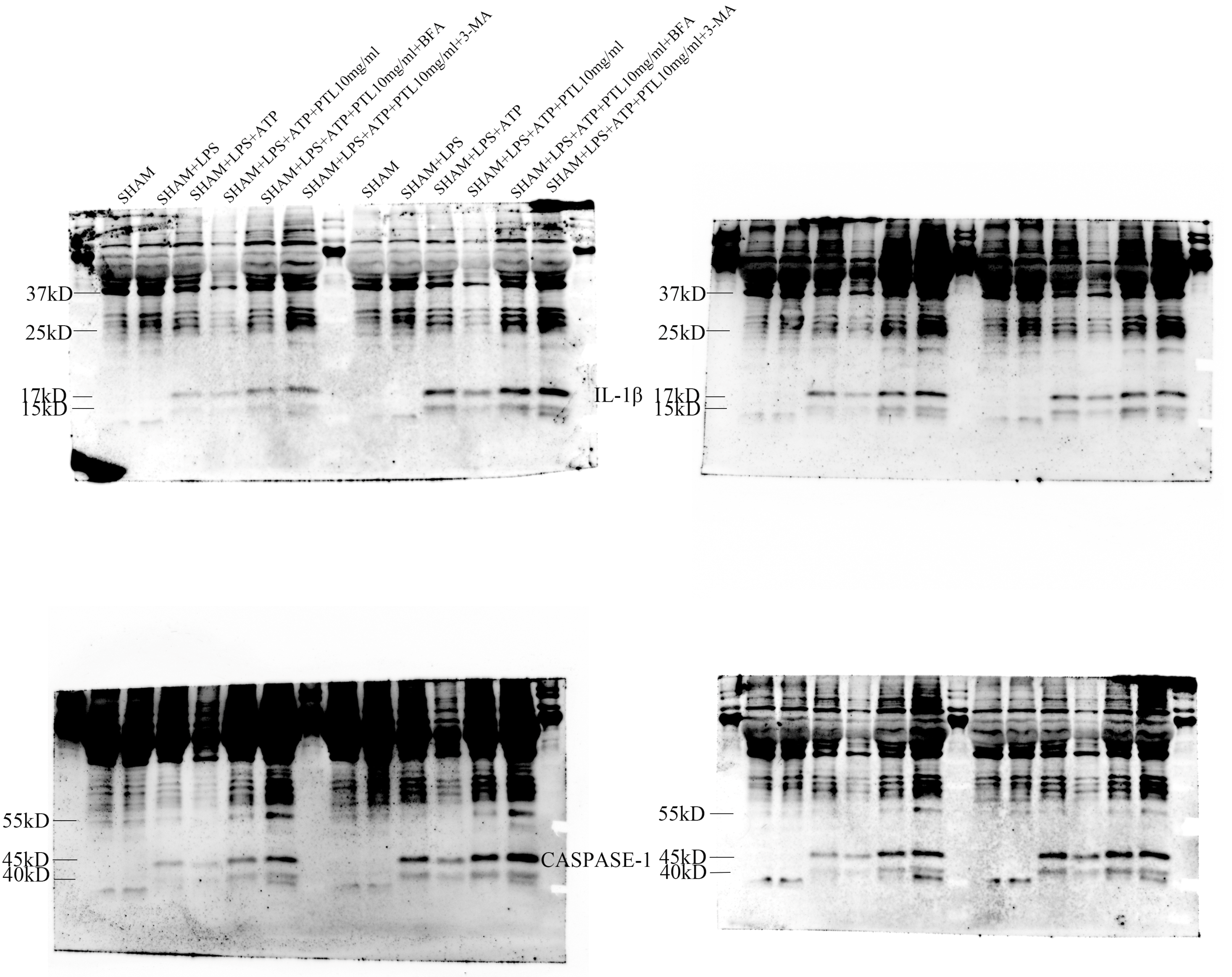

Repeat1 Repeat2 Repeat3 Repeat4

Figure 6.AExample of original western blot

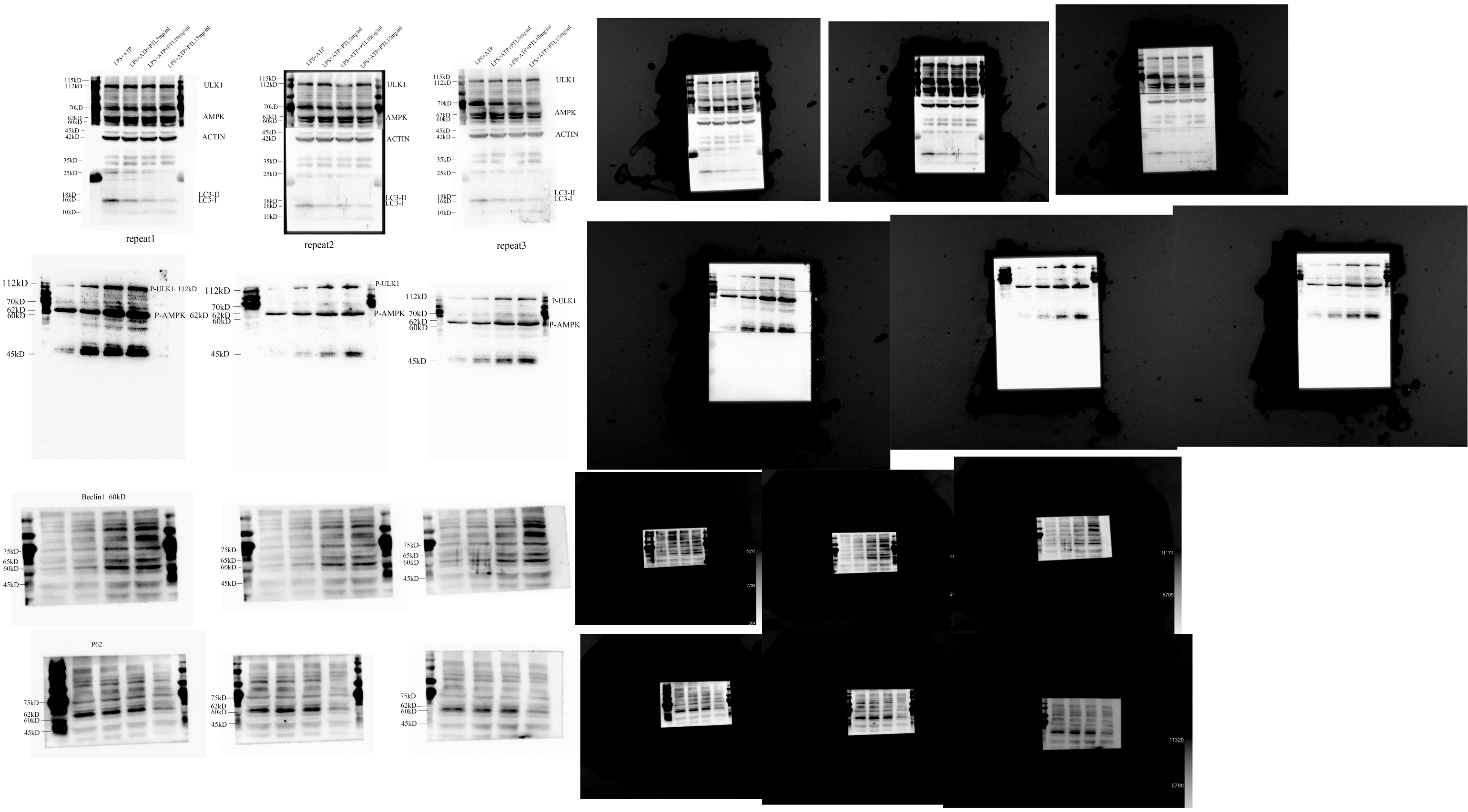

Supplement: Supplementary file 1 — Figure S1-S6 [file ijbms-29-1-90-s001.pdf]
